# Cost analysis of informal care: estimates from a national cross-sectional survey in Sweden

**DOI:** 10.1186/s12913-021-07264-9

**Published:** 2021-11-15

**Authors:** Björn Ekman, Kevin McKee, Joana Vicente, Lennart Magnusson, Elizabeth Hanson

**Affiliations:** 1 Department of Clinical Sciences, Malmö (IKVM), Lund University, Malmö, Sweden; 2 School of Health and Welfare, Dalarna University, Falun, Sweden; 3 Department of Health and Caring Sciences, Linnaeus University, Sweden and Swedish Family Care Competence Centre, Kalmar, Sweden

**Keywords:** Informal care, Costs and cost analysis, Survey, Sweden

## Abstract

**Background:**

Over the past decades, informal care has increased in most OECD-countries. Informal care is costly to caregivers and to society in the form of lost income and direct costs of providing care. Existing evidence suggests that providing informal care affects caregivers’ overall health. However, estimates of the social costs of informal care based on national data on individuals are currently scarce.

**Objective:**

This study contributes to the existing evidence on the costs of informal care by estimating the direct and indirect costs to caregivers using a purposive national household survey from Sweden.

**Methods:**

Adopting a bottom-up, prevalence approach, the direct and indirect costs are estimated using the survey data and the value of working time and leisure time from existing sources.

**Results:**

The results suggest that around 15% of the adult population of Sweden provide informal care and that such care costs around SEK 152 billion per year (around 3% of GDP; USD 16,3 billion; EUR 14,5 billion), or SEK 128000 per caregiver. Around 55% of costs are in the form of income loss to caregivers. The largest cost items are reduced work hours and direct costs of providing informal care. Replacing informal caregivers with professional care providers would be costly at around SEK 193,6 billion per year.

**Conclusions:**

Findings indicate that, even in a country with a relatively generous welfare system, significant resources are allocated toward providing informal care. The costing analysis suggests that effective support initiatives to ease the burden of informal caregivers may be cost-effective.

## Background

Over the past several decades the provision of informal care has increased in most OECD-countries. Estimates of the share of the population that provides informal care vary across countries and range between 10% and 40% of the total population [1, 2]. While definitions of informal care differ somewhat across countries and studies, it is broadly defined as care provided on a voluntary basis by a non-professional individual to a person in need of care, help, or support on a regular basis [3]. The person receiving the care may also receive formal support funded privately or publicly. Informal care may come in different forms, including help with personal care, support with daily chores, and assistance with contacting government agencies. The scope and nature of informal care vary across countries due to demographic factors and also as the scope of formal care varies.

However, regardless of the exact form of informal care, existing evidence suggests that such type of care may impose a significant economic burden on the caregiver and on the broader society [4–7]. For example, the provision of informal care may have an impact on the caregivers’ overall health and on their labor market participation [8, 9]. In addition, including the cost of informal care in economic evaluations of health care interventions and in cost of illness studies has been suggested to be critical for the range of estimates that analysts arrive at as excluding such costs may risk underestimating the true cost and benefits of interventions and burdens of disease [10, 11]. While the economic burden of informal care has been the subject of analyses across a range of countries for some time, there is still a dearth of evidence on the costs of informal care based on national data. Drawing on the findings of a national, cross-sectional survey implemented in 2019, this study seeks to estimate the total costs of informal care in Sweden. The specific objectives are to estimate the indirect costs and the direct informal care costs from the perspective of society. Results from Sweden may be of interest in an international context given the country’s relatively comprehensive social welfare system that includes various types of social care to the elderly and to persons living with some disability [12].

### Review of the current evidence on the costs of informal care

While informal care is common in most countries, the current evidence on the costs associated with such care is limited. Arno and colleagues [13] estimated that in 1997 the total number of care hours in the U.S. was 24 billion valued at around USD196 billion (around USD313 billion in current value). In a more recent study based on time-use survey data from the U.S., Chari and colleagues [14] estimated the total cost of informal care to older adults to be around USD522 billion per year. The difference between these estimates from the U.S. is most likely due to several factors, including divergent types of data, a difference in the definition of informal care and target group, and variance in the approach to costing informal care. While such factors appear to affect a large part of the current literature on informal care, the estimates nevertheless point to substantial societal costs of informal care. In 2001, Langa and colleagues evaluated the scope and cost of informal caregiving for older adults diagnosed with some form of dementia in the U.S. [15]. They found that depending on the severity of the disease, caregivers spend between 8,5 h to 41,5 h per week providing care. This translates into a total cost of informal care for this group of people of USD18 billion per year (equivalent to USD26 billion in today’s value). Based on the findings of Chari and colleagues, the cost of informal care of people with dementia would then make up around 5 % of total informal care of older adults in the U.S.

More recently and using a small sample from two Spanish regions, Oliva-Moreno and colleagues applied various methods to investigate the costs of informal care and its determinants [16]. They found that the annual total costs per caregiver ranged from EUR 14,000 to as much as EUR 80,000 depending on the particular method. Goodrich and colleagues systematically reviewed the economic evaluation literature to investigate how such evaluations have measured the costs of informal care for specific diseases [10]. They found a total of 30 studies that fit their inclusion criteria, 25 of which costed caregivers’ time input. The authors’ main conclusion was that including the cost of informal care in economic evaluations of the cost of providing formal care for specific diseases may alter the implications for funding of treatments. Similar conclusions were reached by Krol and co-authors [17] who systematically reviewed the economic evaluation literature in four disease areas, namely Alzheimer’s disease, cancer, Parkinson’s disease, and rheumatoid arthritis. Overall, only 23% of the cost-effectiveness studies identified in the review included cost estimates of informal care. They noted that inclusion of the cost of informal care may have an impact on overall results and suggested that such costs be included in economic evaluations of health technologies.

One study included in the above review and of relevance to the present study is that of Andersson and colleagues which assessed the cost-effectiveness of two alternative treatments for asthma in a sample of Swedish children [18]. Adopting a societal perspective, they included indirect costs of care, including productivity loss of parents and disrupted sleep. Among other things, they found that both income loss and sleep disruption constitute significant impacts on caregivers. Based on their results it can be seen that these costs represent around 40% of the total costs of asthma treatment.

In a European context, Verbakel and colleagues found that the prevalence of informal care across Europe, i.e. the share of the adult population that provides informal care, varied from 8,2% (in Hungary; different from the rate reported in Baji et al., 2019) to 43,6% in Finland, with an average of 34,3% across all countries [1]. The authors do not report detailed information on the scope of informal caregiving but note that a group identified as “intensive caregivers” provide more than 11 h of informal care per week. This group made up an estimated 7,6% of the total populations, while 3,5% of the populations provided more than 21 h of informal care per week.

The existing evidence on the cost of informal care points at significant amounts of time and resources allocated to informal care. The current study contributes to this evidence by its application of costing analysis to national survey data.

## Methods

To estimate the total societal costs of informal care in Sweden, the study made use of a purposive cross-sectional, observational survey and adopted a prevalence, bottom-up approach where the unit costs are multiplied by the estimated total number of caregivers. The societal perspective involves estimating the resources used to provide informal care across all relevant sections of society, including the individual caregiver, the public sector, and the employer of the caregiver [19, 20]. However, reliable data on resource use by the state and by the employers of caregivers are not readily available. While it is reasonable to assume that these resources may be significant, it is likely that due to the very nature of the activity in question it is the resources used by the caregiver him- or herself that constitute a significant majority of resource utilization.

### Data

The main source of information on key variables and relevant cost items is a recent national survey from Sweden [21]. The survey, which collected information on a range of variables related to the prevalence, scope, and types of activities of providing informal care, was especially designed to collect information about informal care among the general Swedish population. The survey was conducted over the period October 2018 – January 2019 by Statistics Sweden (SCB) on behalf of the research group.

The sampling frame of the survey was the adult population of Sweden (18 years of age or older; *N* = 8,063,051). The sampling frame was stratified by the 21 regions of the country and an equal number of individuals from each stratum was included in the frame. In all, a sample of 30,009 individuals were drawn by stratified, independent random sampling to receive the survey, which could be returned either by surface mail or via a secure webpage. A total of 11,168 persons responded to the questionnaire (response rate 37,3%). All respondents were informed of their right to refrain from participating and that the data would be provided by SCB to the researchers in an anonymized format.

The questionnaire consisted of 29 numbered questions, the last of which provided the opportunity for respondents to give open complementary responses (see https://osf.io/kt3p8/ for details). The questions included both fixed response alternatives and open answers. The survey covered some general background data, information on labor market participation, reception of social benefits and external formal support, and reflections on own experiences of providing informal care. In addition to the survey questions, information on the respondents was added from SCB’s own registers, including sex, age, profession and sector, education, country of birth, residence, and income. The main demographic and socioeconomic indicators are presented in Table 1 for the sample of survey respondents stratified by caregiver status.

**Table 1:** Descriptive statistics of survey data

	Caregiver	Not caregiver	Total
	(*N* = 1798)	(*N* = 9370)	(*N* = 11,168)
**Age, years**			
Mean (SD)	59 (16)	58 (18)	58 (18)
Median (Q1, Q3)	60 (50, 70)	61 (45, 72)	61 (46, 72)
Min, Max	18, 96	18, 100	18, 100
**Gross income, 2017 (SEK)**			
Mean (SD)	569,625 (356,226)	553,832 (360,903)	556,377 (360,184)
Median (Q1, Q3)	499,992 (323,163, 760,268)	485,643 (303,983, 745,655)	488,276 (307,259, 748,358)
Min, Max	0, 2,834,270	0, 5,754,576	0, 5,754,576
**Sex**			
Female	1090 (60.6%)	4892 (52.2%)	5982 (53.6%)
Male	708 (39.4%)	4478 (47.8%)	5186 (46.4%)
**Education level**			
College	759 (42.2%)	3797 (40.6%)	4556 (40.9%)
High School	771 (42.9%)	3929 (42.0%)	4700 (42.2%)
Primary	267 (14.9%)	1620 (17.3%)	1887 (16.9%)
**Employment status**			
Employed	871 (51.3%)	16 (11.5%)	887 (48.3%)
Not employed	215 (12.7%)	20 (14.4%)	235 (12.8%)
Retired	613 (36.1%)	103 (74.1%)	716 (39.0%)
**Born in Sweden**			
Yes	1606 (89.3%)	8431 (90.0%)	10,037 (89.9%)
No	192 (10.7%)	939 (10.0%)	1131 (10.1%)

Source: VANA SE (2019). SD standard deviation. Q1 25th percentile/Q3 75th percentile

Around 15% of respondents were identified as caregivers based on the study’s inclusion criterion: provide care or support on a regular basis to at least one person at least once a month. This inclusion criterion was used as it corresponds with the general definition of a caregiver and with a similar survey conducted in 2012 by the National Board of Health and Social Welfare [22]. In addition, it avoids the risk of overestimating the prevalence of informal care by including persons who provide very little care or do so on an irregular basis. With the exception of sex (relatively more women than men are caregivers) there were no significant differences with respect to demographics and socioeconomics between caregivers and non-caregivers.

In addition to the survey data described above, the costing calculations also used statistics on the prices of a range of items of relevance to the analysis. First, data from Statistics Sweden on wages for certain groups of professionals are used to estimate the replacement cost of informal care. Second, data from Statistics Sweden and from labor union information sites on mean working hours are used to calculate weekly and yearly working hours in Sweden. And third, information on some cost items was collected from the existing evidence base, including the valuation of lost sleep [23].

## Methods

Costing health technologies, a disease, or some activity involves estimating the value of the resources used to implement the technology or activity in question or the economic burden of the particular disease [19]. To arrive at the total societal costs the mean unit cost is multiplied by, in this case, the number of informal caregivers. The main approach of a costing analysis involves three successive steps: *identification* of cost items; *measuring* the costs (or quantification in relevant units); and, finally, *valuing* the resources in monetary terms; here Swedish kronor (SEK; SEK 100 ≈ USD10,13 ≈ EUR 9,2; October 2020). In the case of informal care, the approach also draws on the work of Landfeldt et al. [24], which provides a practical guide for estimating the costs of informal care. In addition, Hoefman et al. [25], and Koopmanschap et al. [20] provide suggestions for how to account for the resources needed to provide informal care.

Consistent with our general prevalence approach, the aim is to estimate the costs associated with informal care over a period of one year. This approach is preferable for long-term conditions, such as a chronic illness [20] and accords with most definitions of informal care as being an activity that extends over a period of time. It also allows for the comparison of the costs of informal care with those of other activities, including the societal costs of diseases and of public programs. Finally, the value of certain intangible resources, such as the impact on overall well-being is not assessed due to lack of reliable data.

### Identification of resources

With respect to identification, the costing analysis assessed resource use across two main domains: the indirect costs of providing informal care and the direct costs of providing care. In turn, the indirect costs of informal care include three separate types of effects, namely those of (i) stopping working due to the need to provide informal care, the impact of (ii) reducing work hours, and the effect on (iii) labor productivity while working. The direct costs of providing informal care include the time cost of providing care, the direct financial costs of care (such as additional costs for transportation, treatments, and other care-related items), and the cost of lost sleep due to providing informal care. A key assumption in the estimation of the cost implications of informal care is that all caregivers need time for sleep and personal activities. To adjust for this requirement, a maximum limit of 126 h per week has been imposed on the total number of care hours that any caregiver can provide (only 14 respondents were affected by this adjustment).

### Quantification and valuation of resources

To measure the cost of lost income or reduced productivity the study employed the human capital approach. This means that the estimated number of hours or share of full-time employment is assessed. Based on the responses on the questions related to the impact of providing informal care on labor input and productivity, the mean values as a share of full-time employment were obtained. In turn, these were multiplied by the total number of respondents who reported such impacts.

The value of one working hour was set at the national mean gross hourly wage plus other labor costs, such as employer and social security contributions (of around 50%).^1^ Consequently, in the third step, these quantities were valued by multiplying the mean number of hours for each item with the mean national hourly gross wage, including employer and social security contributions. In 2018, this value was equal to 263 SEK (www.scb.se/lonestatistik) and represents the opportunity cost of informal care for the sub-sample in question, i.e. those not in retirement.

As noted, the indirect costs of providing informal care were only measured for a sub-sample of the respondents (those who reported such an effect). By separating this sub-set of respondents and by imposing a limit on the total number of hours that can be used for caregiving, the study avoids the risk of double counting. The direct costs of informal care were measured for all the respondents who were identified as caregivers in the survey. The value of the informal care costs was arrived at by multiplying the number of hours with the value of leisure time. Based on existing evidence and on applicable standards for the value of leisure time in Sweden the opportunity cost of leisure time was set at 35% of the national hourly gross wage, net of employer and social security contributions [11, 26, 27]. The direct financial costs were estimated by multiplying the mean amount of out-of-pocket expenditures for the provision of informal care with the number of caregivers.

The value of lost sleep is a question that has received increasing attention over the past decade or so [27]. A recent study using household survey data from OECD-countries suggests that reduced sleep has a range of negative effects and is associated with significant economic losses, including presenteeism [28]. In addition, providing informal care leads to around five minutes of less sleep per day compared to those who do not provide such care (ibid.). Drawing partly on this body of literature, this study makes an assumption that one hour of sleep is equal to the value of leisure, i.e. 35% of the gross hourly wage (net of employer and social security contributions). Given the known effects of disturbed sleep on workplace performance and overall wellbeing this is most likely a conservative assumption.

## Results

With respect to the various cost items identified in step 1 of the costing analysis, Table 2 shows the distribution of the responses stratified by sex for the sample of caregivers. Around 40% of caregivers reported having some level of expenditures due to informal care. The mean amount was estimated at 800 SEK per week. Similarly, around 35% of the sample reported having lost around 30 min of sleep per night due to informal care.

**Table 2:** Summary of cost items by sex

	Female		Male	
	Row %	95% CI	Row %	95% CI
**Caring, number of hours per week**				
Less than 1 h (*n* = 150)	42.15	[28.02–57.69]	57.85	[42.31–71.98]
1–10 h (*n* = 1018)	58.75	[53.35–63.95]	41.25	[36.05–46.65]
11–29 h (*n* = 278)	60.68	[50.14–70.31]	39.32	[29.69–49.86]
30–59 h (*n* = 135)	47.19	[32.60–62.26]	52.81	[37.74–67.40]
60 h or more (*n* = 110)	63.87	[47.14–77.81]	36.13	[22.19–52.86]
Total (*n* = 1691)	56.60	[52.24–60.87]	43.40	[39.13–47.76]
**Total out-of-pocket expenditure (SEK/week)**				
0 (*n* = 697)	60.85	[53.99–67.31]	39.15	[32.69–46.01]
199 (*n* = 508)	51.87	[44.72–58.93]	48.13	[41.07–55.28]
499 (*n* = 281)	58.33	[48.61–67.45]	41.67	[32.55–51.39]
999 (*n* = 124)	51.18	[35.84–66.31]	48.82	[33.69–64.16]
1999 (*n* = 45)	39.32	[21.13–61.05]	60.68	[38.95–78.87]
2999 (*n* = 13)	19.16	[3.17–63.20]	80.84	[36.80–96.83]
3000 (*n* = 10)	77.59	[31.85–96.25]	22.41	[3.75–68.15]
Total (*n* = 1678)	55.90	[51.55–60.17]	44.10	[39.83–48.45]
**Lost sleep (times per week)**				
Not at all (*n* = 1037)	50.67	[45.20–56.12]	49.33	[43.88–54.80]
One night (*n* = 295)	67.46	[58.41–75.37]	32.54	[24.63–41.59]
2–3 nights (*n* = 182)	62.84	[47.87–75.69]	37.16	[24.31–52.13]
4–6 nights (*n* = 74)	56.74	[36.97–74.56]	43.26	[25.44–63.03]
Every night (*n* = 97)	63.51	[45.40–78.46]	36.49	[21.54–54.60]
Total (*n* = 1685)	56.06	[51.72–60.31]	43.94	[39.69–48.28]
**Stopped working**				
No (*n* = 1742)	55.45	[51.17–59.65]	44.55	[40.35–48.83]
Yes (*n* = 56)	70.28	[45.76–86.89]	29.72	[13.11–54.24]
Total (*n* = 1798)	55.96	[51.75–60.09]	44.04	[39.91–48.25]
**Affected employment**				
No (*n* = 993)	57.16	[51.68–62.46]	42.84	[37.54–48.32]
Sick leave (*n* = 18)	50.04	[18.05–82.00]	49.96	[18.00–81.95]
Less than half (*n* = 21)	53.48	[22.70–81.81]	46.52	[18.19–77.30]
Half (*n* = 17)	79.87	[45.33–94.99]	20.13	[5.01–54.67]
More than half (*n* = 100)	57.80	[42.45–71.77]	42.20	[28.23–57.55]
Total (*n* = 1149)	57.43	[52.37–62.35]	42.57	[37.65–47.63]
**Affected ability to work (%)**				
No (*n* = 745)	56.16	[49.82–62.31]	43.84	[37.69–50.18]
10 (*n* = 189)	55.76	[44.35–66.59]	44.24	[33.41–55.65]
25 (*n* = 106)	68.62	[50.91–82.18]	31.38	[17.82–49.09]
50 (*n* = 49)	59.07	[34.10–80.10]	40.93	[19.90–65.90]
More than 50 (*n* = 26)	47.48	[20.50–76.02]	52.52	[23.98–79.50]
Total (*n* = 1115)	57.18	[52.04–62.16]	42.82	[37.84–47.96]
**Received support from local government**				
No (*n* = 1481)	57.47	[52.88–61.93]	42.53	[38.07–47.12]
Yes (*n* = 317)	49.39	[39.36–59.46]	50.61	[40.54–60.64]
Total (*n* = 1798)	55.96	[51.75–60.09]	44.04	[39.91–48.25]

Source: VANA SE (2019)

The data suggest that there are some differences between men and women with respect to these costing indicators. For example, for those who reported having had their work affected in some way, that impact appear to have been stronger among women compared with men.

Applying the methods described above to the current survey data and using relevant values obtained from other sources, the analysis finds that the total costs of informal care to caregivers in Sweden was around SEK 152 billion in 2018 (Table 3). This is approximately 3,15% of total gross domestic product of some SEK 4,8 trillion in the same year. The cost per caregiver is around SEK 128000 per year on average. The cost of informal care in Sweden is largely driven by the indirect costs of lost income and lost productivity of caregivers. These losses make up some 55% of the total estimated cost of informal care.

**Table 3:** Total annual societal costs of informal care in Sweden, 2018 (SEK)

Domain	Cost Item	Costs	Share of total costs, %
**A. Lost income/productivity**			
A.1	Cessation of work	21,040,045,347	14
A.2	Reduction of work hours	42,443,895,514	28
A.3	Lost productivity while working	20,009,265,028	13
*Sub-total*		*83,493,205,889*	*55*
**B. Direct cost of caregiving**			
B.1	Caregiving time	40,998,299,353	27
B.2	Out-of-pocket financial costs	22,529,180,952	15
B.3	Lost sleep	5,114,705,508	3
*Sub-total*		*68,642,185,814*	*45*
**Total societal costs**		**152,135,391,703**	

Source: Authors’ own calculations

The single largest cost item is the costs related to the need to reduce work hours at around SEK 42 billion or 28% of total costs. Of almost the same value is the time that caregivers spend providing informal care. At around SEK 41 billion per year, this is 27% of the total costs of informal care. Direct financial costs due to added expenses for caregiving amounted to around SEK 22 billion or 15% of the total costs. Finally, the cost of lost sleep is estimated at around SEK 5,1 billion or 3 % of total costs.

### Replacement cost analysis

The analysis above shows that informal care imposes significant indirect and direct costs on the part of caregivers. While providing informal care to a close relative or acquaintance also has benefits in terms of a sense of accomplishment and satisfaction, a relevant policy question to pose is one that asks what it would cost to replace the informal caregivers with formal care providers, such as assistant nurses. Formal professional care is usually provided by assistant nurses at the municipal level. The mean gross wage of an assistant nurse was SEK 28400 per month in 2018 (www.scb.se/lonestatistik). The total labor cost of one assistant nurse is 28,400 × 1,5 (employer and social security contributions) which is equal to SEK 42600 per month or SEK 267 per hour. Based on these numbers, the total gross replacement cost would be 725 million hours multiplied by SEK 267 which is equal to SEK 193,6 billion. The replacement cost would thus exceed the estimated total costs to informal caregivers.

However, from a societal perspective the net cost of replacing informal carers with formal caregivers would be considerably less as the gross costs would be offset by the value of the time freed-up for informal caregivers and broader benefits that would entail. Depending on their ability to go back to work and to increase their productivity while working this effect could be considerable if also health benefits and the impact on overall wellbeing is taken into consideration [4, 7, 8].

### Sensitivity analysis

The above analysis is based on a set of assumptions that underpin the calculations. For example, the mean wage of caregivers was assumed to be equal to the national mean gross wage in Sweden in 2018 of around SEK 31900. The income data provided on the participants from Statistics Sweden suggest that the mean gross wage of caregivers and non-caregivers were SEK 26000 and SEK 25500, respectively. Using the mean gross wage of caregivers from the survey to estimate the costs of providing informal care would reduce the total costs to around SEK 140 billion.

In addition, the estimates do not take into account the value of any support from the municipality or other source received by the caregivers. Some respondents indicated that they had received such support. However, estimating the value of any such support is difficult as some of it is normally provided in kind. The omission of these benefits is unlikely to result in any major misrepresentation of the costs of informal care on a national level.

Disregarding the effect of lost sleep on caregivers would reduce the total costs by another SEK 5,1 billion. On the other hand, the value of leisure is difficult to identify. Using the value suggested by Verbooy and colleagues [29] of EUR 16 per hour would increase the total costs to around SEK 218,1 billion. The range of cost estimates would then be between SEK 140 billion and SEK 218 billion per year for informal care in Sweden.

## Discussion

At almost 150 billion SEK (or 3 % of total GDP), the societal cost of informal care represents a significant economic burden on society. It is the caregiver him- or herself that bears the brunt of these costs. To put the cost estimate of informal care in perspective, it is noted that the cost of informal care is around one third of the expenditures on health care in Sweden (around SEK 480 billion per year) and half of all spending on education (SEK 309 billion per year). However, the annual cost of informal care is significantly larger than the societal costs of some common conditions in Sweden: Diabetes Type-2 (around SEK 80 billion per year); mental ill health (SEK 75 billion); and cardiovascular diseases (SEK 61,5 billion of which SEK 18 billion is for informal care). Compared with these broad public expenditure programs and common diseases, the cost of informal care to caregivers in Sweden is relatively large.

The result of this analysis is comparable with the findings from other studies. For example, the total per capita cost of informal care of SEK 128000 per year is equivalent to the lower end of the result of Oliva-Moreno and colleagues based on Spanish data noted above [16]. The Swedish cost of informal care also appear to be lower than that of the U.K. where the cost per caregiver was found to be around GBP 19300 (approx. SEK 223000) in a study from 2015 [30, 31]. While the differences between these estimates may be partly due to differences in the definition of informal care and variations in analytical approaches, they all point at significant amounts.

The survey data allow for an estimate of the total number of hours of informal care in Sweden per year. Based on the total number of caregivers and the mean number of hours of informal care per week, the analysis suggests that a total of 725 million hours of informal care is provided in Sweden each year. To replace informal care with professional care would cost a total of 193,6 billion SEK. It is unlikely that all or even a large share of informal care could be replaced by professional carers in the short- or even the medium-term. First, it would imply a prohibitively large amount of financing, the vast majority of which would have to come from increased local taxation. Second, even if the funding could be mobilized it is unclear whether there would be sufficient personnel available to take up such work. Sweden, like most other OECD-countries, is facing significant challenges in finding and retaining staff across most sectors of society [4, 32]. And third, there would also be added costs to any effort to replace informal carers with professional carers in the form of training and wage increase costs as a result of the surge in demand. However, given the observed differences in wage levels between men and women and the fact that formal caregivers are predominantly women, any increase in demand for such professionals may affect the relative wage differences between the sexes.

The findings of this study are relevant also to other countries. Sweden, along with some other northern European countries, has a relatively comprehensive social welfare system where the provision of formal care to those who need it is generous in comparison with many other countries. However, the findings suggest that even in such a context, the costs of informal care are large. It would therefore be reasonable to assume that such costs may be even larger in other countries with less generous social welfare systems. As such, the findings point to important policy issues across most OECD-countries that may have an impact on labor market participation rates, equality between men and women, and overall quality of life.

The main limitation of this study is the exclusion of resources used by the local government and by employers of caregivers to enable the estimation of the true societal costs of informal care. While these costs are most likely real, they are difficult to estimate in a reliable manner. However, it is unlikely that their exclusion skews the results in any substantial way as the main costs of informal care fall on the caregiver him- or herself. The study also omits the value of any benefits received for similar reasons. Finally, it is also a limitation that no convincing estimates of intangible costs, such as the effects on caregivers’ health and overall wellbeing can be provided. For example, providing informal care may be stressful, which could have a direct negative health impact and an indirect effect on the caregiver’s productivity. These effects may be substantial, and their inclusion would most likely increase the cost estimates by some share.

## Conclusions

The results of this study contribute to the existing evidence base on the implications of the provision of informal care and is relevant to future policy development in the area of social wellbeing. By employing national survey data, the study is able to provide the first estimates of the costs of informal care at the national level in a European context. It also applies recognized methods to the data to ensure the ability to compare the findings with other studies employing similar methods. Further studies are needed to understand the cost of informal care also to other parts of society, including local municipalities and employers and of the intangible effects on informal carers.

## Data Availability

The datasets used and/or analysed during the current study available from the corresponding author on reasonable request.

## Footnotes

1 Using the median hourly wage did not change the estimates in any material way.
